# Causal relationship between metabolic dysfunction-associated fatty liver disease and endotoxin biomarkers: A Mendelian randomization study

**DOI:** 10.1097/MD.0000000000042311

**Published:** 2025-05-16

**Authors:** Jingwen Kong, Xixi Han, Chao Wei

**Affiliations:** a Jining Medical University, Jining, China; b Beijing University of Chinese Medicine, Beijing, China.

**Keywords:** lipopolysaccharides, LPS-binding proteins, Mendelian randomization, metabolic dysfunction-associated fatty liver disease

## Abstract

Although the relationship among lipopolysaccharides (LPS), LPS-binding proteins, and metabolic dysfunction-associated fatty liver disease (MAFLD) is widely studied, no conclusive evidence is available. In this study, we used mendelian randomization (MR) to study the causal relationship of LPS, LPS-binding proteins, and MAFLD. Using bidirectional two-sample MR method, we evaluated data from the genome wide association study; for this analysis, nonalcoholic fatty liver disease (NAFLD), liver fat percentage, and other metabolic syndromes were employed as outcomes. Furthermore, MR analysis mainly involved the inverse variance weighted method. Heterogeneity and pleiotropy tests were also conducted. LPS was found to have a causal relationship with NAFLD, obesity, high density lipoprotein cholesterol, and TG levels. Furthermore, TG levels and LBP had significant causal relationships. This study mainly concluded that LPS is a risk factor for NAFLD, obesity, high density lipoprotein cholesterol, and TG, corroborating it’s the LPS role in MAFLD pathogenesis. Hence, optimizing the gut microbiota using proper diet, probiotics, or fecal microbiota transplantation may help to reduce inflammation and (IR), thereby improving lipid and glucose metabolism disorders. Although a causal relationship between TG and LBP was observed, further studies are required to determine a specific mechanism.

## 1. Introduction

Nonalcoholic fatty liver disease (NAFLD) is characterized by an excessive deposition of fat in liver cells; this fat accumulation is not due to alcohol consumption or other clear causes. The incidence rate of NAFLD is approximately 38% and is showing increasing trend in recent years.^[1,2]^ In 2022, 134 medical groups recognized the need to rename NAFLD as metabolic dysfunction-associated fatty liver disease (MAFLD) because the latter term emphasizes the importance of metabolic disorders in the occurrence and development of fatty liver, and hence can better guide clinical targeted management.^[3,4]^ In case of liver steatosis, MAFLD can be diagnosed if obesity or type 2 diabetes mellitus (T2DM) is present; however, if the body weight is within normal clinical range, >2 risk factors (increased waist circumference, hypertension, hypertriglyceridemia, low high density lipoprotein cholesterol (HDL-c) level, abnormal fasting blood glucose (FBG), insulin resistance and chronic inflammatory) of metabolic disorder should be present.^[5,6]^ Due to adverse changes in lifestyle, there is a considerable increase in the occurrence frequency of various metabolic syndromes, such as obesity, T2DM, and atherogenic dyslipidemia (AD). Such metabolic disorders not only pose risk for MAFLD, but also may lead to serious liver complications and cardiovascular diseases, increasing mortality risk for such patients.^[7–9]^

The importance of increased lipopolysaccharide (LPS) levels in the liver during NAFLD is extensively studied. LPS, which is a component of Gram-negative bacterial outer membrane and a potent endotoxin, can be accumulated in the liver by forming LPS-binding protein (LBP) complexes, in turn initiating a series of inflammatory reactions in the liver.^[10]^ LPS penetrates the bloodstream through the intestinal mucosa, instigating inflammatory reactions by engaging the Toll-like receptor 4 (TLR4). Upon arriving at the liver, LPS stimulates TLR4-mediated inflammation within hepatocytes, which plays a role in the progression of NAFLD among overweight and obese individuals.^[11]^ Meanwhile, LPS is an important factor in regulating lipid metabolism and can increase TG synthesis in the liver.^[12]^ LPS can also lead to oxidative stress and mitochondrial damage, playing an important role in the process of liver inflammation.^[13]^ Experimentally reduced liver LBP levels, whether in standard conditions or non-obesogenic factors, intensify liver inflammation, fibrosis, and oxidative stress in nonalcoholic steatohepatitis (NASH) in mouse models.^[14]^ Elevated serum LPS and LBP levels in rat model of NAFLD associated with obesity.^[15]^ Further, high serum LPS and LBP levels predicted high level of obesity in children and indicated risk for the development of metabolic syndrome in adolescents.^[16]^ However, contrast results were also reported; individuals with obesity showed increased LBP levels but a negative correlation with T2DM, NAFLD, and nonalcoholic steatosis (NASH). According to these results, LBP may serve as a protective factor, mitigating the adverse metabolic consequences associated with obesity.^[17]^ Contrasting results have also been observed in terms of circulating LPS levels in patients with T2DM. Since these data were obtained from studies with different designs, such as cross-sectional studies, population surveys, and cohorts, confounding factors and reverse causal effects are inevitable.^[18]^

Mendelian randomization (MR) is a study of mendelian inheritance based on the random allocation of alleles from parents to offspring; this process is equivalent to that of random allocation of participants in randomized controlled trials. MR is advantageous because it is not affected by traditional confounding factors and satisfies temporal rationality. The basic idea is to use genetic variations strongly correlated with exposure factors as instrumental variables to infer causal effects between exposure and outcome.^[19,20]^ This study aimed to employ MR to understand the bidirectional causal relationship among various metabolic disorders related to MAFLD and LPS, and LBP. We also explored the mechanism of interaction between LPS, LBP, and MAFLD.

## 2. Materials and methods

### 2.1. Study design

**Figure 1.**
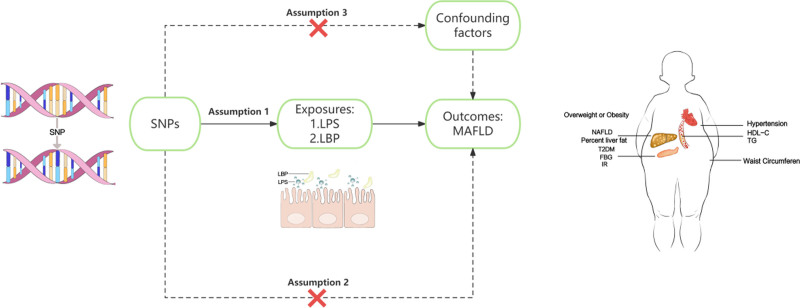
Graphical Abstract: the 3 assumptions of Mendelian randomization analysis.

The genome wide association study (GWAS) database was used to perform a two sample bidirectional MR analysis. We followed the following 3 MR hypotheses: single nucleotide polymorphism (SNP) is strongly correlated with exposure, SNP is independent of confounding factors that affect exposure and outcomes, and SNP can only affect outcomes through exposure rather than other pathways. The three major hypotheses and variables of this study are shown in Figure 1. After satisfying the three major hypotheses of Mendelian randomization, we conducted correlation setting, independence setting, and statistical strength setting, calculated P-values and F-values, and then conducted heterogeneity testing and sensitivity analysis to obtain funnel plots and forest plots.

### 2.2. Data sources

NAFLD, liver fat percentage, and factors related to metabolic disorders were considered as outcomes. The large-scale GWAS meta-analysis (included the UK Biobank, eMERGE, FinnGen cohorts, and Estonian Biobank) was the source for NAFLD data. The diagnostic criteria were based on EHR codes except conditions, such as alcoholic liver disease, hepatitis, and inbound errors of metabolism.^[21]^ Considering the important association between visceral fat and NAFLD, liver fat percentage was also considered; the data were obtained from the UK Biobank abdominal magnetic resonance imaging. Overweight data were obtained fom the European population of 51 studies conducted by the Genetic Investigation of ANthropometric Traits (GIANT).^[22]^ Obesity data were from European ethnicities, including 4793 patients with obesity and 209884 with no obesity. UKB, GERA, and DIAGRAM databases were the sources for T2DM data, with the majority being European and a small portion from Pakistan.^[23]^ Waist circumference and hypertension data were from the UKBiobank database (European descent). Data on HDL-c were from a longitudinal study of British households, and blood samples were collected for testing and analysis.^[24]^ Triglyceride (TG) level data were from the UK Biobank.^[25]^ FBG data from 58070 non-diabetes European individuals recruited from 29 MAGIC studies.^[26]^ Insulin resistance (IR) data were also from individuals with no diabetes in MAGIC study.^[27]^ Serum LPS activity data were from Finnish Twin Cohort (Adult Finnish twins), FinnDiane (type 1 diabetes and unclassified diabetes), and FINRISK (Finnish population); the serum LPS activity was measured by using either spectrometry-based method or via the methods of limulus amebocyte lysate (LAL).^[28]^ LBP is a cohort from a Finnish study in which participants were recruited for the general surgery in the UK region. Based on proteomic analysis, the fasting plasma EDTA levels were measured.^[29]^ All databases utilized are publicly available and were approved by the ethics committees of original studies. Furthermore, all participants in those studies provided consent for participation. Hence, additional ethics approval or consent was not required. Table 1 lists features all databases employed.

**Table 1:** The basic characteristics of GWAS data.

Trait	GWAS ID	Consortium	Population	Sample size (case/control)	PMID
NAFLD	ebi-a-GCST90091033	eMERGE + UK Biobank + FinnGen + Estonian Biobank	European	778,614 (8434/770,180)	34841290
Percent liver fat	ebi-a-GCST90016673	UK Biobank	European	32,858	34128465
Overweight	ieu-a-93	Genetic Investigation of ANthropometric Traits (GIANT)	European	158855	23563607
Obesity	finn-b-E4_OBESITYNAS	NA	European	214677 (4793/209,884)	NA
T2DM	ebi-a-GCST006867	DIAbetes Genetics Replication and Meta-analysis (DIAGRAM) + Genetic Epidemiology Research on Adult Health and Aging (GERA) + UKB	European + Pakistan	655,666 (61,714/1178)	30054458
WC	ukb-b-9405	UKBiobank	European	462,166	NA
Hypertension	ukb-b-14057	UKBiobank	European	462,933 (119,731/343,202)	NA
HDL-C	ebi-a-GCST005058	The United Kingdom Household Longitudinal Study	European	9796	28887542
TG levels	ebi-a-GCST90025957	UKBiobank	European	437,532	34226706
FBG	ebi-a-GCST005186	Glucose and Insulin-related traits Consortium (MAGIC)	European	58,074	22581228
IR	ebi-a-GCST005179	Glucose and Insulin-related traits Consortium (MAGIC)	European	37,037	20081858
LPS	ebi-a-GCST90032674	FinnDiane, FINRISK and Finnish Twin Cohort	European	11,296	34668383
LBP	ebi-a-GCST90019429	The Fenland study	European	10,708	33328453

FBG = fasting blood glucose, IR = insulin resistance, LBP = lipopolysaccharide-binding protein, LPS = lipopolysaccharide, NAFLD = nonalcoholic fatty liver disease; T2DM = type 2 diabetes, TG = triglyceride levels, WC = waist circumference.

### 2.3. Selection of inverse variances

Screening of SNPs (with *P* < 5 × 10^−8^ from the GWAS) strongly correlated with exposure factors (significance adjusted to *P* < 5 × 10^−6^ when the number of SNPs was small).^[30,31]^ To remove the linkage imbalance among SNPs, satisfying *r*^2^ = 0.001 and kb = 10,000 was essential. After deleting palindromic SNPs, we used PhenoScanner website to exclude SNPs associated with confounding factors. We eventually calculated the F value (equation 1) and *R*^2^value (equation 2) for each SNP, excluding weak variables with F < 10.^[32]^

$$\begin{array}{l} \left(F=\left(\frac{R^{2}}{1-R^{2}}\right)\left(\frac{n-k-1}{k}\right)\right) \end{array}$$ (1)

$$\begin{array}{l} \left(R^{2}=2\left(1-MAF\right)MAF{\left(\beta \right)}^{2}\right). \end{array}$$ (2)

### 2.4. Statistical analysis

MR analysis involved inverse variance weighted (IVW) supplemented by weight median (WM), MR Egger, simple mode, and weighted mode. The MRPRESSO package (V1.0) was used; it detects outlier SNPs and then eliminates them for re-analysis and result comparison. We selected SNPs with *P* < 5 × 10^−8^ or *P* < 5 × 10^−6^, When a threshold of *P* < 5 × 10^−6^ was applied to remove linkage disequilibrium (*r*^2^ = 0.001, kb = 10,000). With F-values greater than 10, and then removed the palindrome sequence. Sensitivity analysis of MR results was performed using Cochran’s Q-test results (*P* < .05 indicated gene heterogeneity). MR-Egger intercept analysis was used to test gene pleiotropy (*P* < .05 indicated horizontal pleiotropy) and leave-one-out method was used to test result stability by removing SNPs. All statistical analyses were conducted using the TwoSampleMR package (V 0.6.1) in R version 4.3.3.

## 3. Results

### 3.1. Inverse variance screening results

We first used a significance threshold of *P* < 5 × 10^−8^ or *P* < 5 × 10^−6^ and obtained six SNPs related to LPS and LBP each. 18 and 19 SNPs related to LPS and LBP, respectively, were obtained. On the PhenoScanner website, no SNP related to confounding factors was obtained. For observed SNPs, the F values were > 10, indicating strong correlation. Subsequently, we extracted the information of these SNPs in MAFLD, removed the palindrome sequence, and removed outlier SNPs using MRPRESSO, and finally obtained inverse variances for the MR analysis. The inverse variance screening process in case of reverse MR is described in Table S1, Supplemental Digital Content, https://links.lww.com/MD/O880.

### 3.2. Bilateral MR of LPS and MAFLD

**Figure 2.**
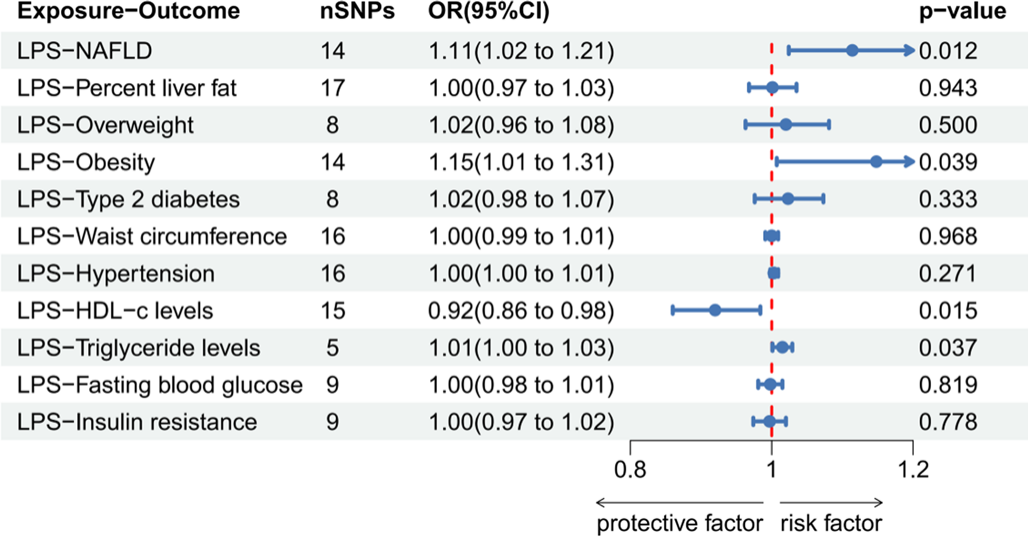
The IVW method to determine whether LPS is a risk factor for MAFLD. IVW = inverse variance weighted, LPS = lipopolysaccharides, MAFLD = metabolic dysfunction-associated fatty liver disease.

**Figure 3.**
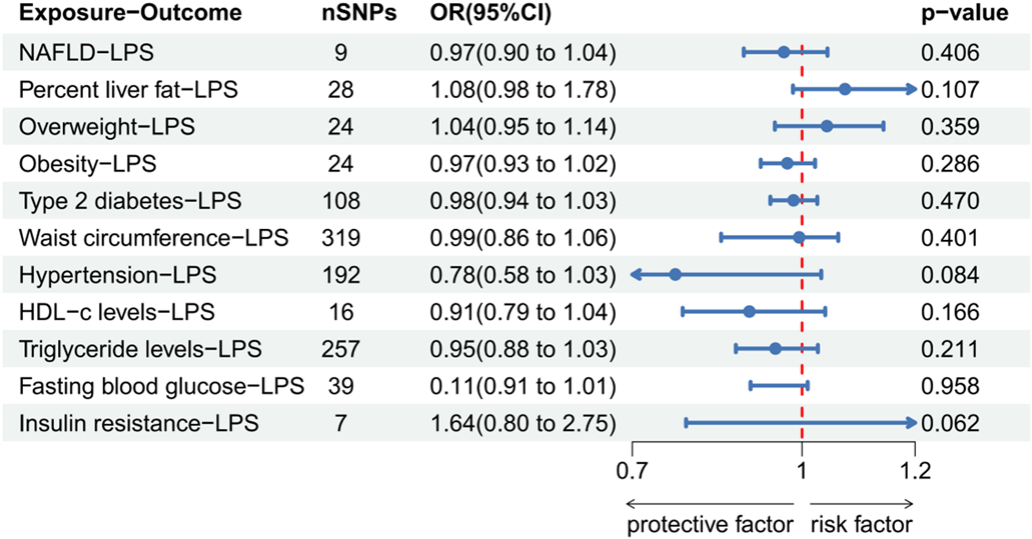
The IVW method to determine whether MAFLD is a risk factor for LPS elevation. IVW = inverse variance weighted, LPS = lipopolysaccharides, MAFLD = metabolic dysfunction-associated fatty liver disease.

LPS was found to have a causal relationship with NAFLD (odds ratio [OR]: 1.114, 95% confidence interval [CI]: 1.024–1.211, *P* = .012), obesity (OR: 1.148, 95% CI: 1.007–1.308, *P* = .039), HDL-c (OR: 0.920, 95% CI: 0.860–0.984, *P* = .015), triglyceride levels (OR: 1.015, 95% CI: 1.001–1.029, *P* = .037); hence, increasing LPS levels led to an increased incidence of NAFLD and obesity, decreased levels of HDL-c, and increased TG levels (Figure S1, Supplemental Digital Content, https://links.lww.com/MD/O881 the Scatter plot, funnel plot, forest plot, and leave-one-out sensitivity analysis in MR analysis). Heterogeneity was also observed between LPS levels and the occurrence of obesity (Cochran’s Q = 23.365, Q *P* value = .038) Notably, no significant heterogeneity was observed between LPS and other results. Moreover, in the MR-PRESSO global test, no horizontal pleiotropy was observed in the overall instrumental variables, and the outlier corrected test showed no outlier SNPs. Other tests, such as the MR Egger intercept test that indicated no pleiotropy and the leave-one-out sensitivity analysis (based on SNP positioning), also demonstrated robustness of conclusions. The reverse MR analysis showed that NAFLD, liver fat percentage, overweight, obesity, HDL-c, FBG, and IR (analysis significance threshold of *P* < 5 × 10^−6^) as well as T2DM, waist circumference, hypertension, and TG levels (analysis significance threshold of *P* < 5 × 10^−8^) were not risk factors for elevated LPS levels. Table 2 shows the results of heterogeneity, pleiotropy, and MR-PRESSO tests, whereas Figures 2 and 3 show forest maps.

**Table 2:** Heterogeneity test, Pleiotropy test and MR-PRESSO test.

Exposure	Cochran’s Q *P* value	egger_intercept	MR-PRESSO global test *P* value
LPS-NAFLD	.200	0.548	.302
LPS-Percent liver fat	.10	0.51	.168
LPS-Overweight	.175	0.636	.387
LPS-Obesity	.038	0.576	.058
LPS-Type 2 diabetes	.707	0.464	.673
LPS-Waist circumference	.047	0.783	.078
LPS-Hypertension	.00	0.1608361	.022
LPS-HDL-c levels	.387	0.306	.493
LPS-Triglyceride levels	.576	0.317	.431
LPS-Fasting blood glucose	.9569845	0.8969849	.987
LPS-Insulin resistance	.9746196	0.2985206	.94
LBP-NAFLD	.9087747	0.3668788	.873
LBP-Percent liver fat	.4626318	0.2126812	.392
LBP-Overweight	.87988	0.5661833	.974
LBP-Obesity	.8266659	0.3229417	.904
LBP-Type 2 diabetes	.08268243	NA	NA
LBP-Waist circumference	1.77E-09	0.4748781	<.001
LBP-Hypertension	.000830803	0.7266216	.031
LBP-HDL-c levels	.370	0.439	.402
LBP-Triglyceride levels	.000641233	0.2758084	.065
LBP-Fasting blood glucose	.7800669	0.900061	.817
LBP-Insulin resistance	.6633514	0.5850178	.919
NAFLD-LPS	.529	0.278	.41
Percent liver fat-LPS	.559	0.347	.267
Overweight-LPS	.556	0.201	.642
Obesity-LPS	.309	0.277	.241
Type 2 diabetes-LPS	.237	0.162	.3
Waist circumference-LPS	.965	0.958	.848
Hypertension-LPS	.088	0.058	.174
HDL-c levels-LPS	1.505 × 10-6	0.173	<.001
Triglyceride levels-LPS	.009	0.329	<.001
Fasting blood glucose-LPS	.807	0.163	.285
Insulin resistance-LPS	.537	0.874	.234
NAFLD-LBP	.053	0.62	.135
Percent liver fat-LBP	.35	0.736	.162
Overweight-LBP	.231	0.971	.559
Obesity-LBP	.879	0.597	.911
Type 2 diabetes-LBP	.463	0.098	.214
Waist circumference-LBP	.104	0.016	.117
Hypertension-LBP	.016	0.686	.021
HDL-c levels-LBP	.618	0.784	.787
Triglyceride levels-LBP	.0013	0.52	.002
Fasting blood glucose-LBP	.012	0.119	.285
Insulin resistance-LBP	.005	0.817	.016

HDL-c = high density lipoprotein cholesterol, LBP = lipopolysaccharide-binding protein, LPS = lipopolysaccharide, NAFLD = nonalcoholic fatty liver disease.

### 3.3. Bidirectional MR of LBP and MAFLD

**Figure 4.**
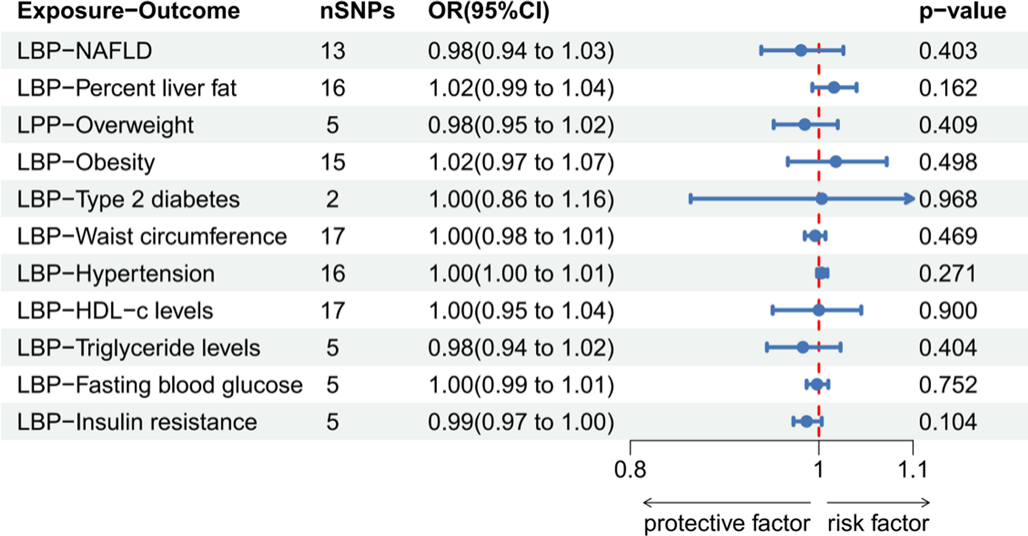
The IVW method to determine whether LBP is a risk factor for MAFLD. IVW = inverse variance weighted, LBPs = LPS-binding proteins, MAFLD = metabolic dysfunction-associated fatty liver disease.

**Figure 5.**
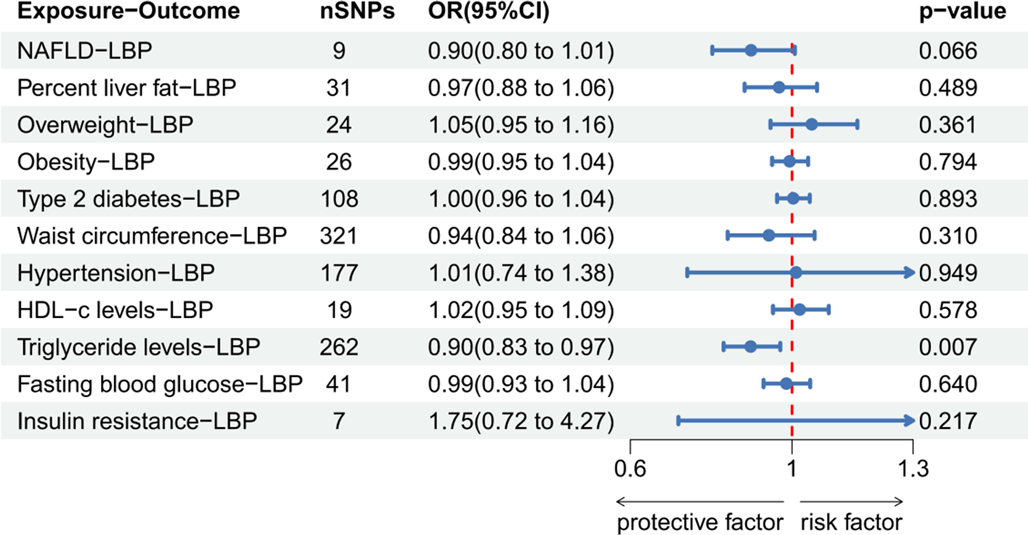
The IVW method to determine whether MAFLD is a risk factor for LBP elevation. IVW = inverse variance weighted, LBPs = LPS-binding proteins, MAFLD = metabolic dysfunction-associated fatty liver disease.

LBP was not found to be a risk factor for MAFLD-related diseases. In the reverse MR analysis (NAFLD, liver fat percentage, overweight, obesity, HDL-c, FBG, and IR [analysis significance threshold of *P* < 5 × 10^−6^] as well as T2DM, waist circumference, hypertension, and TG levels [analysis significance threshold of *P* < 5 × 10^−8^]), a significant causal relationship was noticed between TG levels and LBP (an increase in TG levels correlated with a decrease in LBP [OR: 0.898, 95% CI: 0.831–0.971, *P* = .007]). (Figure S2, Supplemental Digital Content, https://links.lww.com/MD/O882 the Scatter plot, funnel plot, forest plot, and leave-one-out sensitivity analysis in MR analysis of LBP and TG) Based on the results of Cochran’s Q-test (significant heterogeneity [Q = 334.97, *P* = .0013]) and MR-PRESSO global test (*P* value = .002), presence of horizontal pleiotropy was established. However, outlier corrected test showed no outlier SNPs (MR-Egger intercept = 0.001, Egger intercept *P* value = .52, no horizontal pleiotropy). Figures 4 and 5 show relevant forest maps.

## 4. Discussion

This study identified a causal association among LPS, LBP, NAFLD, and related metabolic disorders; LPS is a risk factor for NAFLD, obesity, HDL-c levels, and TG levels. Furthermore, LBS is a protective factor against elevated TG levels. Based on the best of our knowledge, this is the first study in which MR analysis was formed to determine the association between LPS, LBS, and NAFLD.

Previously, a meta-analysis of 43 studies showed that blood LPS is a good marker for NAFLD and its staging, and blood LPS levels can also serve as a marker for evaluating intestinal permeability in NAFLD.^[33]^ Heng Yuan et al found that LPS biosynthesis is higher in Asian patients with MAFLD compared with that in patients with no MAFLD.^[34]^ Previous experiments conducted in rats also suggested that LPS can promote the occurrence and development of fatty liver.^[35]^ The journey of LPS to the liver initiates from the gut where dysbiosis of gut microbiota damages the intestinal barrier function, subsequently leading to a leakage of LPS to the bloodstream. LPS utilizes the portal vein and TLR4 receptors to reach the liver and triggers Kupffer cells to produce various inflammatory cytokines and cause liver damage.^[36,37]^ It has been demonstrated that LPS increases the lipid accumulation in liver cells by inhibiting DNA methyltransferase 3B (DNMT3B), thereby reducing CIDEA promoter methylation. The present study clarifies the fact that an initial imbalance in gut microflora and bacterial translocation in bloodstream lead to increase in the rate of NAFLD.^[38]^ Hence, to control MAFLD pathogenesis, the role of gut microbiota should be considered. By implementing methods, such as diet, use of probiotics, and fecal microbiota transplantation, it may be possible to reduce inflammation and IR, improve lipid and glucose metabolism disorders, and thereby reduce MAFLD incidence.^[39]^

The present study showed that an increase in LPS levels also raises the incidence of obesity and lipid disequilibrium (decrease in HDL-c levels and increase in TG levels). Consistent with these results, a previous prospective study that conducted a 6-year follow-up of 393 adolescents found that serum LPS predicted obesity, high TG levels and low HDL-c levels.^[16]^ Another study conducting a meta-analysis showed a negative correlation between LPS and high HDL-c levels.^[40]^ Several animal studies have also shown similar results; LPS was shown to decrease HDL-c levels and increase TG levels in mice.^[41–43]^ A previous cross-sectional study also demonstrated a close association of LPS with visceral fat in 41 participants with normal BMI; the TG level of the high visceral fat group was significantly higher than that of the low visceral fat group.^[44]^ Inflammation is an important pathogenesis of hyperlipidemia and a driving factor connecting obesity and gut microbiota. Excessive inflammation can lead to metabolic diseases, such as obesity and hyperlipidemia.^[45]^ When the intestinal barrier is damaged, LPS enters the circulation and activates the TLR4-NF-κB-IL-1b/IL-6/TNF-ɑ axis to release inflammatory cytokines. Inflammation reduces lipid metabolism in the liver and adipose tissue, leading to obesity, T2DM, and hyperlipidemia. Studies have shown that low-dose LPS can cause lipid metabolism disorders in humans.^[46–48]^ Interestingly, a research has observed that the vitamin D levels in sheep exhibit significant fluctuations between the summer and winter months, yet detect no significant alterations during an immune challenge with LPS endotoxin.^[49]^ A meta-analysis has found that supplementation with vitamin D may lower the incidence of SARS-CoV-2 infections necessitating ICU admission and mechanical ventilation.^[50]^ Given the vital role that vitamin D plays in sustaining peak immune function, it is imperative to conduct additional research into the correlation between fluctuations in vitamin D levels and LPS to better understand how they may influence metabolic disruptions.

LBPs are acute phase reactive proteins, with a high affinity for the lipid A portion of LPS, and hence these proteins can display proinflammatory and anti-inflammatory actions. The present study showed a causal relationship between TG levels and LBPs (decreased LBP levels are related to increased TG levels). No correlation of LBPs was found with NAFLD, T2DM, obesity, waist circumference, and FBG. Using multifactorial logistic regression analysis, a previous cross-sectional study demonstrated that LBPs are independent protective factors for T2DM and NAFLD. In addition, LBPs were positively correlated with waist circumference and negatively correlated with FBG. Hence, LBPs can promote favorable glucose metabolism to avoid NAFLD.^[17]^ Another study suggested that LBPs are biomarkers of obesity-related IR.^[51]^ Although LBP levels were found to be significantly elevated in patients with NAFLD in another study, LBP level was imposed as an independent risk factor for NAFLD. In addition, LBP was significantly positively correlated with TG, FBG, and IR.^[52]^ Furthermore, in a cross-sectional study on the independent correlation between LBP levels and liver fat fraction and liver volume in 99 adolescents with obesity showed that LBP levels can be used as biomarker for predicting high NAFLD risk in adults.^[53]^ Previous epigenetic studies conducted in LBP knockout NAFLD animal models demonstrated decreased inflammation (characterized by decreased levels of IL-6, TNF-ɑ), intensified lipid accumulation, and more severe NAFLD. LBP deficiency was mainly attributed to the transcriptional activation of C/EBP-β and SCD, leading to excessive lipid deposition and exacerbating high fat diet (HFD)-induced NAFLD. This also indicates that HFD feeding induced NAFLD models can lead to liver with LBP deficiency.^[54]^ These can, in part, explain the findings of the present study where HFD was found to promote TG levels; hence, increased TG levels or the condition of hypertriglyceridemia, can decrease LBP levels.^[55]^

In a case-control study involving 386 patients diagnosed with nonalcoholic steatohepatitis (NASH), the analysis revealed that NASH constitutes an independent risk factor for venous thromboembolism (VTE), with a particular association to deep vein thrombosis.^[56]^ This alerts clinicians that such patients frequently necessitate hospitalization and intensive management, encompassing anticoagulation therapy. In recent years, there has also been increasing attention to antiplatelet therapy for NAFLD.^[57]^ Experimental studies have indicated that treatment with aspirin and thienopyridine may alter the progression of liver steatosis, potentially decreasing the incidence of NAFLD and liver fibrosis.^[58]^ Some meta-analyses revealed that both prophylactic anticoagulation and the use of compression stockings were effective in reducing the incidence of venous thromboembolism (VTE).^[59,60]^

The main strengths of the present study include high evidence level, effective avoidance of bias, and assessment of reverse causality. Furthermore, the GWAS (database used in the present analysis) had participants with European ethnicities, and hence, we avoided regional and ethnic biases, whereas the exposure and outcome variables came from different study cohort participants, effectively reducing sample overlap. This study also had a few limitations. First, heterogeneity was observed in SNPs between LPS and obesity, without observation of any horizontal pleiotropy. Significant heterogeneity was also detected in SNPs between TG and LBP but with MR-Egger intercept of 0.001. At the same time, the Egger intercept *P* value was .52, indicating no horizontal pleiotropy. Second, this study included only European population. Hence, for other ethnic population, these results will have to be extrapolated. Further studies will be required to verify the findings of this study in other ethnic populations.

## 5. Conclusion

This bidirectional two-sample MR study showed that LPS is a risk factor for NAFLD, obesity, TG, and HDL-C. However, MAFLD did not cause an increase in LPS levels. Targeting the gut microbiota and intestinal barrier may become a highly promising therapy for preventing and treating MAFLD. Although TG levels and LBP levels were found to be negatively correlated, further research is needed to understand the interaction mechanism of LBP and MAFLD.

## Acknowledgments

We appreciate all the GWAS data provided by https://www.ebi.ac.uk/gwas.

## Author contributions

**Conceptualization:** Xixi Han.

**Data curation:** Xixi Han, Chao Wei.

**Formal analysis:** Xixi Han.

**Investigation:** Jingwen Kong, Xixi Han.

**Methodology:** Jingwen Kong, Xixi Han.

**Project administration:** Jingwen Kong, Xixi Han, Chao Wei.

**Resources:** Jingwen Kong, Xixi Han.

**Software:** Jingwen Kong, Xixi Han.

**Supervision:** Xixi Han, Chao Wei.

**Validation:** Chao Wei.

**Writing – original draft:** Jingwen Kong.

**Writing – review & editing:** Jingwen Kong, Chao Wei.

## Supplementary Material
