# Supplementary material for: Causal relationship between metabolic dysfunction-associated fatty liver disease and endotoxin biomarkers: A Mendelian randomization study
Source: Medicine (Baltimore). 2025 May 16;104(20):e42311. doi: 10.1097/MD.0000000000042311 (PMC12091621; doi:10.1097/MD.0000000000042311)
Supplement: Supplementary file 2 [file medi-104-e42311-s002.pdf]

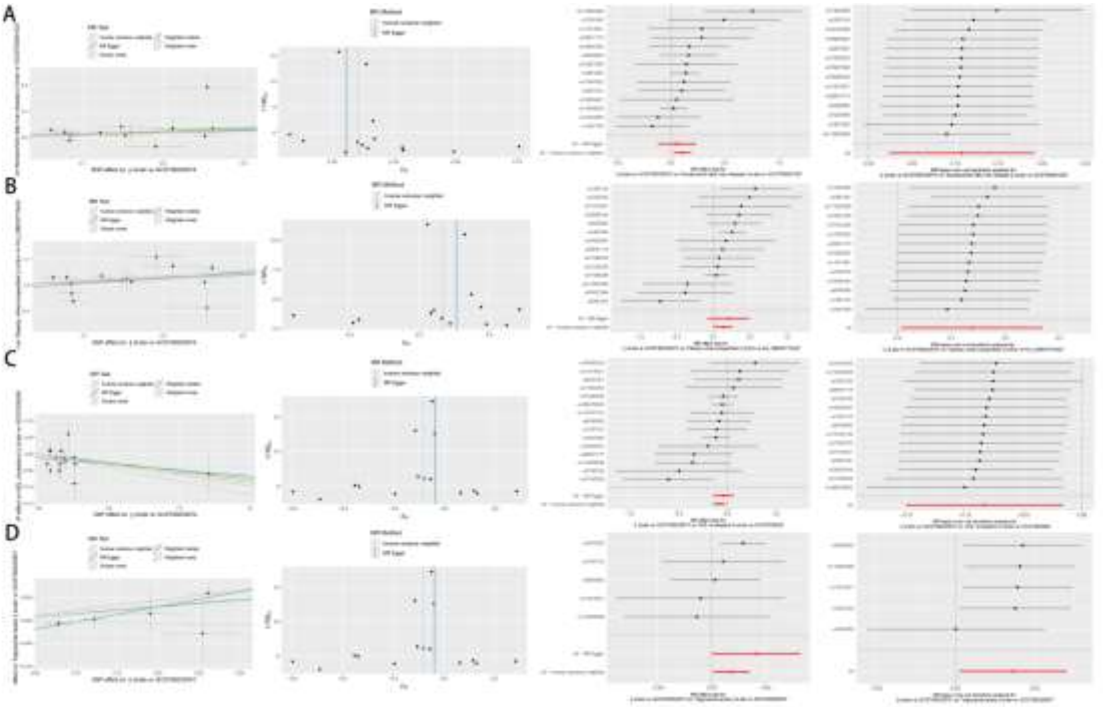

(A) Scatter plot, funnel plot, forest plot, and leave-one-out sensitivity analysis in MR analysis of LPS and NAFLD.

(B) Scatter plot, funnel plot, forest plot, and leave-one-out sensitivity analysis in MR analysis of LPS and obesity.

(C) Scatter plot, funnel plot, forest plot, and leave-one-out sensitivity analysis in MR analysis of LPS and HDL-c.

(D) Scatter plot, funnel plot, forest plot, and leave-one-out sensitivity analysis in MR analysis of LPS and TG.
